# Post-Hypoxic Myoclonus and Deep Brain Stimulation. Experience from a Small Patient Cohort and Literature Review Highlighting Variable Outcomes

**DOI:** 10.5334/tohm.1171

**Published:** 2026-07-07

**Authors:** Tiziana De Santis, Alon Y. Mogilner, Michael Pourfar

**Affiliations:** 1 Marlene and Paolo Fresco Institute for Parkinson’s and Movement Disorders, NYU Langone Health, New York, US; 2 Department of Neurology, IRCCS Humanitas Research Hospital, Rozzano, Milano, IT; 3 Department of Neurosurgery, NYU Langone Health, New York, US

**Keywords:** Lance-Adams, deep brain stimulation, post-hypoxic myoclonus

## Abstract

Lance Adams Syndrome (LAS), or chronic post-hypoxic myoclonus (PHM), is a rare and disabling condition which occurs in the aftermath of a diffuse hypoxic brain injury, usually when patient regains consciousness. Due to its refractoriness to antiepileptics, deep brain stimulation (DBS) has been attempted over the past fifteen years.

We describe three patients who underwent Globus Pallidus interna (GPi)-DBS at the NYU Langone Department of Neurosurgery. Despite varying stimulation parameters and contact combinations, none achieved substantial improvement of action and negative myoclonus or regained the ability to ambulate unassisted.

We compared our results with previous reports. Our literature review identified ten cases of LAS treated with DBS since 2010. The target was GPi in 8/10 patients, the ventralis intermedius (VIM) nucleus in one, and the subtalamic nucleus (STN) in one. Varying degrees of improvement were reported, with some evidence suggesting that long pulse width and lower frequency might be more effective. However, most reports provided only partial Unified Myoclonus Rating Scale (UMRS) scores, with missing information on rest and reflex myoclonus as well as negative myoclonus.

Due to the paucity of prior reports and the limited generalizability of existing evidence, the efficacy of DBS in LAS has yet to be demonstrated. Further case reports, both positive and negative, supported by neurophysiological measures, may help bridge this knowledge gap.

## Introduction

Post-hypoxic myoclonus is a form of myoclonus that occurs in patients who have experienced a diffuse hypoxic brain injury, usually in the context of cardiopulmonary arrest (CPA). Lance-Adams syndrome is the chronic type of PHM that typically develops days to weeks after a hypoxic brain injury [1]. It is characterized by both positive and negative myoclonus, reflecting the mixed cortical/subcortical dysfunction. The myoclonic jerks in LAS can be observed at rest, action and induced with stimulus and often co-exist with ataxia and seizures. The treatment of LAS is challenging. Clonazepam, valproic acid, and levetiracetam [2] are first line treatments, usually used in combination [3]; however, up to 50% of cases prove medication-refractory [4]. In refractory cases, L-5-hydroxytryptophan, piracetam, lacosamide, zonisamide, agomelatine, cannabidiol [5], perampanel [6], and sodium oxybate [7] have been employed with variable results [3,8,9]. DBS has been utilized as a treatment of last resort in refractory cases of LAS based on the well documented therapeutic efficacy reported in other myoclonic syndromes such as myoclonus-dystonia (MD) [10,11]. The data on DBS outcomes for LAS, however, remains limited to a small number of patients and generally highlights positive results. We present three relatively unfavorable outcomes from our institution along with a review of reported literature to highlight the heterogeneous nature of DBS-responsiveness in this challenging condition.

## Methods

We reviewed data on patients diagnosed with LAS who underwent DBS at NYU Langone Medical Center between January 2010 to October 2025. Clinical history, myoclonus phenomenology, surgical details including stereotactic coordinates and stimulation parameters, and medications at each follow-up visit along with subjective reports of patient/caregiver and neurologist, were documented. Clinical assessments, UMRS and videos were included when available.

Informed consent was obtained for videotaping. Analysis of the data was approved by the Ethics Committee of NYU IRB (S21-01610).

We additionally reviewed the Pubmed database for previous publications of similar cases. A Medline search was conducted using the following search strategy: (hypox*[TiAb]OR anox*[TiAb]) AND (myoclon*[TiAb] OR Lance Adams[TiAb]) AND (DBS[TiAb]OR Deep brain Stimulation[TiAb]). Supplementary figure 1 reports the selection flow-chart.

We collected data related to: (a) age and gender; (b) etiology of myoclonus; (c) time of onset of PHM after the anoxic injury; d) time interval between PHM onset and DBS; e) DBS target and stimulation parameters; f) follow-up duration; g) pre- and post-surgery UMRS; e) pre- and post-surgery antiepileptic regimen. Supplementary figure 2 shows the neuroanatomical reconstruction of each patient’s leads.

## Results

### Case descriptions

#### Case 1

A 25-year-old male had a witnessed pulseless electrical activity (PEA) arrest from massive bilateral pulmonary emboli. He was resuscitated after 30–40 minutes, and treated with thrombolysis and hypothermia protocol. Shortly after his intubation, he developed decerebrate posturing and abnormal jerking movements of his arms and legs. Brain and spine MRI were unremarkable, and video EEGs demonstrated intermittent central polyspike and slow wave activity against a normal electrographic background. After weaning off sedation, he eventually regained consciousness and was cognitively intact. His abnormal movements persisted and were characterized by large amplitude, action-induced myoclonus that interfered with all activities of daily living. After two months of rehabilitation, he was able to eat with weighted utensils, drink with a straw and use the toilet with assistance. Dressing, bathing, and self-care remained difficult on account of the action-induced myoclonus. He could not stand or take steps without support due to severe negative myoclonus. He was treated with clonazepam, valproate, levetiracetam, sodium oxybate and zonisamide with modest benefit. Routine EEG performed 24 months after the hypoxic event showed centrally predominant cortical irritability. Twenty-four months after the hypoxic event, he underwent uneventful bilateral GPi-DBS with Abbott directional leads. He experienced a promising microlesional effect but returned to baseline by the time of initial programming. Monopolar review of the left GPi demonstrated good tolerability while the right caused mild dysarthria ventrally with a suggestion of benefit dorsally. Initial parameters were C+1– 2.0 V/60 usec/130 Hz (Left GPi) and C+11– 2.0 V/60 usec/130 Hz (Right GPi). Little improvement was reported despite interim increases in amplitude. Over the following 48 months, multiple parameters were explored including interleaving and multi-contact stimulation with variable pulse widths (PW) as well as with high and low frequency (**supplementary table 1**). He reported modest benefit on low frequency and high PW settings, seemingly able to stand more easily despite persistent myoclonus (**supplementary video**). His rest myoclonus abated, while action myoclonus improved only marginally, and negative myoclonus remained unchanged (Table 1). Further supporting some efficacy, he was able to discontinue zonisamide and reduce the valproate dose. Unfortunately, any benefit was soon overwhelmed by worsening medical status independent of – though exacerbated by – persistent myoclonus. He developed cachexia leading eventually to J-tube placement and became unable to stand without two-person support. At last follow-up, he described the benefit from DBS as being “around 5%.”

**Table 1:** DBS coordinates and parameters and UMRS scores in our cohort.


	LEAD TIP COORDINATES	INITIAL SETTINGS	MOST-EFFECTIVE SETTINGS	UMRS PRE-DBS	UMRS POST-DBS

**Patient 1**	L Lead: X: 20.2 Y: 1.8 Z: –5.5 R Lead: X: 20.8 Y: 0.1 Z: –6.0	L GPi: C+2–: 2.0 V/60 usec/130 Hz R GPi: 10+11– 2.0 V/60 usec/130 Hz	L Gpi: 2.0 V/130 µs/30 Hz R Gpi: 10+11–: 2.0 V/130 µs/30 Hz	Rest myoclonus: 12 Action myoclonus: 70 Negative myoclonus severity score: 3	Rest myoclonus: 0 (100%) Action myoclonus: 61 (–13%) Negative myoclonus severity score: 3

**Patient 2**	L Lead: X: 19.0 Y0.8 Z: –4 R Lead: X: 19.2 Y: 0.3 Z: –3.5	L GPi: C+2 1.5 V/60 usec/130 Hz; R GPi: C+11– 1.5 V/60 usec/130 Hz	L Gpi: C+1– 1.0V/130usec/30 Hz R Gpi: 8+9– 1.0 V/130 usec/30 Hz	Rest myoclonus: 21 Action myoclonus: 11 Negative myoclonus severity score: 1	Rest myoclonus: 7 (–65%) Action myoclonus: 6 (–45%) Negative myoclonus severity score: 1

**Patient 3**	L Lead: X: 18.5 Y: 0.4 Z: –5.7 R Lead: X: 18.5 Y: 0.3 Z: –5.3	LGPi 0–1+: 2.0V/ 90us/130 Hz R GPi 8–9+: 2.0 V/90us/130 Hz	–	–	–


**Abbreviations:** DBS: Deep Brain Stimulation; GPi: globus pallida interna; L: left; R: right; UMRS: Unified Myoclonus Rating Scale.

#### Case 2

A 56-year-old man developed PHM following elective cervical discectomy complicated by a postoperative hematoma leading to CPA. He had a prolonged stay in the ICU with refractory seizures. When he regained consciousness, he exhibited generalized involuntary muscle jerks worsened by voluntary movements. EEG showed generalized polyspikes often correlated with myoclonus, interpreted as mixed cortical and subcortical myoclonus. He underwent extensive rehabilitation and regained significant function but had persistent myoclonus that limited walking and activities of daily living. He was trialed on multiple combinations of antiepileptics and experienced only temporary improvement with sodium oxybate.

The patient underwent uneventful bilateral GPi-DBS using Abbott DBS directional leads 42 months after the hypoxic event. Initial programming revealed good tolerability with some capsular limitations in the 4.0 mA range (speech, hand pulling), more apparent ventrally. He was started on monopolar stimulation using C+2– 1.5 V/60 usec/130 Hz (Left GPi) and C+11– 1.5 V/60 usec/130 Hz (Right GPi). He initially appeared to benefit with a reduction of truncal and action myoclonus allowing him to perform activities like using utensils and walking more easily. However, after one year of continuous stimulation the perceived benefit wore off. Multiple programming changes were undertaken (**supplementary table 2**) but never resulted in a clear benefit save for modest improvement with low frequencies and long PW, which also waned over time (**supplementary video**). There was a 65% improvement in the UMRS score for resting myoclonus, while action myoclonus improved by 45% and negative myoclonus remained unchanged (Table 1). Due to the lack of significant perceived benefit from the stimulation, he often turned the DBS off after 3 years on account of persistent action and negative axial myoclonus.

#### Case 3

An 18-year-old male developed PHM following spontaneous pneumothorax and cardiac arrest. He received two rounds of cardiopulmonary resuscitation for 5 minutes and was intubated. He was unconscious and soon after developed involuntary movements. EEG showed stimulus-sensitive, high-amplitude, irregular 5–6 Hz EMG myoclonus artifact, appearing diffusely with a frontal predominance without clear cortical cerebral correlate, compatible with myoclonus without seizure activity. Repeat MRI showed abnormalities involving bilateral putamina and caudate nuclei without associated diffusion restriction, which resolved on follow-up (figure 3). He was diagnosed with LAS, characterized by severe impairment due to positive and negative myoclonic jerks, with slow improvement over time. He partially benefited from treatment with valproic acid, levetiracetam and sodium oxybate. He underwent uneventful bilateral GPi-DBS using Medtronic non-directional leads 24 months after the hypoxic event (Table 1). Monopolar review demonstrated somewhat low capsular thresholds in the 2V range using high frequency bilaterally, but good tolerability using bipolar settings with no immediately evident benefit. He was started on 0–1+: 2.0V/ 90 us/130 Hz (L GPI) and 8–9+: 2.0V/90us/130 Hz (R GPi). He reported little improvement despite multiple subsequent adjustments including low and high frequencies and a variety of contacts and variations (supplementary table 3). Despite the lack of apparent improvement with stimulation, his myoclonus trended better over the years. He turned DBS off for prolonged periods due to perceived speech difficulties without noting any change in his myoclonus. Due to lack of perceived benefit he decided to remove DBS seven years after implantation, which was performed without complication.

## Literature review

Our search strategy retrieved ten prior cases of LAS treated with DBS (Table 2) [12,13,14,15,16,17,18,19,20,21]. The first case dates to 2010. The age at onset of PHM ranged from 23 to 79 years. Six of ten patients were males. In all cases, save for one perinatal anoxia case, PHM followed acute CPA. In some cases, the precise etiology of CPA, the time involved in resuscitation and the latency to development of PHM were not reported. The time from the occurrence of PHM to DBS implantation ranged from 5 to 384 months. The selected DBS target was the GPi in 8/10 patients, with one VIM and one STN case reported. Follow-up after DBS implantation ranged from 6 to 60 months; only three patients were followed for more than 12 months. Table 2 provides detailed information about prior cases. Table 3 lists the UMRS scores.

**Table 2:** Demographic and clinical data and DBS parameters from prior studies and our cohort.


STUDY	AGE/GENDER	ETIOLOGY	FOLLOW-UP DURATION AFTER DBS (MONTHS)	TIMETO DBS (MONTHS)	DBS TARGET/ELECTRODE	DBS PARAMETERS (CONTACTS: AMPLITUDE/PW/FREQUENCY)

**Kobayashi et al**. [13]	36M	Perinatal hypoxia	24	384	BL VIM	R: 1–3+/210 μs /135 Hz L: 1–3+ 210 μs /135 Hz

**Yamada et al**. [14]	71 M	CPA after pulmonary embolism	10	10	Left Gpi Awake, sterotactic	L: 1–2+1,8 V/450 μs/130 Hz

**Asahi et al**. [15]	54M	CPA following obstruction of the tracheal tube by phlegm	6	12	BL Gpi Awake, sterotactic	R: 1– 2+ 2.5 V/60 μs/125 Hz L: 0–1+ 2.0 V/60 μs/125 Hz

**Ramdhani et. al**. [20]	23M	CPA after asthmatic attack	6	36	BL Gpi Awake, sterotactic	R: 3-c+: 2.8 V/90 μs/130 Hz L: 1–2–3–C+: 2.5 V/60 μs/130 Hz

**Gao et al**. [12]	33F	CPA after drowning	12	5	BL Gpi Asleep, iMRI guided	R: 1–2 –C+: 3.2 V/60 μs/180 Hz L: 1–2 –C+:3.2 V/60 μs/180 Hz

**Mure et al**. [19]	79M	CPA after carotid endarterectomy	24	12	BL Gpi Awake, sterotactic	R: 3+2–1–, 2.5 mA/212 μs/110 Hz L: 3+2–1–, 4.25 mA/237 μs/110 Hz

**Kim et al**. [18]	34F	CPA after drowning	60	36	BL Gpi Awake, sterotactic	R: C+0– 2,5 V/130 μs/35 Hz L: C+1– 2,5 V/130 μs/35 Hz

**Ozturk et al**. [16]	40F	CPA after cesarean delivery	36	156	BL Gpi Awake, sterotactic	3 V/60 μs/160 Hz

**Tharp et al**. [17]	38M	CPA	9	NA	BL STN General anesthesia	L: C+ 2b,c- 1.0 mA/90/130 Hz R: C+ 10b- 1.7 mA/90 us/130 Hz

**Kaur et al**. [21]	56F	CPA after suicide attempt	6	24	BL Gpi adaptative Under anesthesia	Monopolar R: C+2–: 3.5 mA/90 µs/130 Hz L: C+9–: 3.5 mA/90 µs/130 Hz.

**Patient 1**	27M	CPA after pulmonary embolism	52	24	BL Gpi General anesthesia	L: 2.0 V/130 µs/30 Hz; R: 10+11–: 2.0 V/130 µs/30 Hz

**Patient 2**	56M	CPA after thyroid artery rupture	46	42	BL Gpi General anesthesia	L: C+2– 1.5 V/60 µs/130 Hz, R: C+11– 1.5 V/60 µs/130 Hz

**Patient 3**	18M	CPA after spontaneous bilateral PNX	84	24	BL Gpi General anesthesia	L: 0–1+: 2.0 V/90 µs/130 R: 8–9+: 2.0 V/90 µs/130 Hz


**Abbreviations:** BL: bilateral; CPA cardiopulmonary arrest; DBS: Deep brain stimulation, GPi: globus pallida interna; L: left, PNX: pneumothorax; PW: pulse width; R: right.

**Table 3:** DBS outcomes of prior cases.


	UMRS SCORES PRE-DBS			UMRS SCORES POST-DBS		
	
	REST	ACTION	REFLEX MYOCLONUS	REST	ACTION	REFLEX MYOCLONUS

Kobayashi et al. [13]	–	14	–	–	11(–21%)	–

Yamada et al. [14]	24	52	–	6 (–75%)	15 (–71%)	–

Asahi et al. [15]	8	25	5	0 (100%)	5 (–80%)	0 (–100%)

Ramdhani et al. [20]	75	52	0	0 (100%)	32 (–39%)	0

Gao et al. [12]	61	2	–	40 (– 34%)	0 (100%)	–

Mure et al. [19]		90	–	–	24 (–73%)	–

Kim et al. [18]	32	80	17	52 (–35%)	3 (–90%)	8 (–53%)

Ozturk et al. [16]	30	112	13	24 (–20%)	103 (–8%)	11 (–15%)

Tharp et al. [17]		29	–	–	28 (–4%)	–

Kaur et al. [21]	18	104	0	12 (–33%)	43 (–59%)	0


**Abbreviations:** DBS: deep brain stimulation, UMRS: Unified Myoclonus Rating Scale.

### Stimulation parameters

The parameters used in reported GPi DBS cases varied considerably. Single monopolar and multiple monopolar contacts were used in some cases [12,18,20,21] whereas bipolar was utilized in others [14,15,19]. In one case the selected contacts were not reported [16]. Amplitudes varied from 1 to 4.25 V and pulse widths from 60 to 550 us. High frequency (>100 Hz) was employed in nine cases, whereas low frequency (35 Hz) was employed in one [18]. Details of the programming approach were not reported in most cases.

Varying degrees of improvement were reported (Table 3). The best outcome is that of Asahi et al [15], using interleaved bipolar, high frequency stimulation. Gao et al. [12] reported 100% reduction of action myoclonus and a 34% reduction of resting myoclonus using a double monopolar configuration. In one case reported by Kaur et colleagues [21], local field potential sensing was implemented, tracking alpha and gamma bands, with rest myoclonus correlated to activity at 11 and 50 Hz. The authors reported 33% improvement in rest myoclonus, 66% improvement in negative myoclonus, and 50% improvement in the global disability score based on the UMRS.

When VIM was targeted [22] the strongest effect was noted with bipolar stimulation and long PW. At the 24-month follow-up, the severity of action myoclonus of the arm measured by the UMRS score was reduced by 21% on the left and by 100% on the right.

Subthalamic nucleus was targeted in a patient with concurrent generalized tonic-clonic seizures stemming from the sensory-motor cortex and LAS [17]. Monopolar configuration with cathodic stimulation was employed bilaterally. No objective changes were seen although the patient reported subjective improvement.

## Discussion

We report outcomes of three patients who experienced little to modest benefit following GPi-DBS for PHM. These results are at variance with the small number of positive outcomes reported in the literature and thus warrant consideration when deciding on and discussing DBS for patients with LAS.

The rationale of the use of GPi-DBS in LAS lays in its reported efficacy on rest and action myoclonus in patients with myoclonus dystonia [10,11,23]. Myoclonic jerks in MD related to mutations in the SGCE gene are believed to be subcortical in nature [24]. This is in contrast to the myoclonus observed in PHM, which is often of a more complex, mixed nature. The subcortical myoclonus of LAS has been classified as reticular (stemming from brainstem), in contrast with the subcortical myoclonus seen in MD [25,26]. The distinction between cortical and subcortical origins is not always reported in PHM DBS cases, as dedicated neurophysiological assessments were rarely performed and EEG alone is often insufficient to determine the source of myoclonus (supplementary table 4) [27,28] When multimodal neurophysiologic assessment was conducted, there was strong evidence that myoclonus originates in the cortex [27].

For cortically-generated myoclonus, the STN is a potentially attractive target given reports of STN DBS’s efficacy in progressive myoclonic epilepsy [29]. However, the only case of STN-DBS for PHM showed no objective improvement of myoclonus after the implantation [17], highlighting the challenge in assessing DBS cause and effect in PHM.

Comprehensive neurophysiological evaluation to better characterize the neural generator of myoclonus might provide further insights to help better inform target selection [30].

It is conceivable that different outcomes relate to different programming parameters. We were, however, unable to convert any non-responder to a responder by shifting the field of stimulation, changing from high to low frequency or adjusting other parameters in any number of ways. Another consideration involves optimal target selection. The GPi has been the most consistently selected target and the one selected for all three of our center’s patients.

The only case of VIM-DBS [22] showed some discrepancies compared with the others, as the myoclonus was a consequence of perinatal injury, involved only the upper limbs, and began four years after the hypoxic event. Furthermore, the patient underwent DBS 384 months after the onset of LAS, representing the longest interval reported between the hypoxic event and surgical intervention.

We did observe an improvement in rest myoclonus but this did not translate to improved quality of life. The reasons for the lack of notable improvement in our small cohort are unclear but do not appear to obviously relate to stereotactic targeting or programming. We cannot rule out the possibility that slight alterations in lead positioning or a different approach to programming might have elicited a better response but neither seemed overtly at odds with those reported in positive responders. Nor did we find evident baseline differences compared with previously published patients, although data regarding the precise etiology of the hypoxic event were lacking in some cases, as well as information on the duration of resuscitation and latency to development of PHM, suggesting a probable variability in the severity and the extent of anoxic brain injury.

Time interval between the hypoxic event and DBS implantation may also be an important factor, as one of our patients underwent DBS after 24 months and two after 48 months. In all but two prior cases, time interval was below 36 months. The worst reported outcome was Otzurk et colleagues [16] and was likely attributed to the long latency between the hypoxic event and DBS surgery (156 months).

The severity and the length of the hypoxic event, the nature of myoclonus (subcortical vs cortical; pure myoclonus vs myoclonus combined with ataxia and seizures) and the difficulty of its assessment should also be considered. Age at onset of myoclonus, disease duration, and length of follow-up have been shown to correlate significantly with relative changes in UMRS scores in MD [11], but comparable data are lacking in PHM. Finally, the inconsistency in UMRS scales across studies should be taken into consideration, since in most cases only partial data was reported, with missing information on rest and reflex myoclonus as well as negative myoclonus, which is one of the most important determinants of functioning, as it reflects the capacity to ambulate.

Our study has limitations, some of which are inherent to small case series that restrict generalizability. We were unable to model the volume of tissue activated (VTA) given the large number of combinations of programming parameters that were tested.

Additionally, the UMRS was not available for patient 3, but the lack of positive outcomes in all three cases was corroborated by both the treating team and the patients, reflecting their lived experience with DBS and PHM.

## Conclusion

The efficacy of DBS on PHM requires further investigation. While a small number of case reports support its use and potential for benefit, these three cases highlight the variable and often disappointing outcomes that are, perhaps, common but not frequently reported.

We acknowledge that the generalizability of these findings is limited, given the small number of patients reported both in our case series and in existing literature. However, we believe that awareness of such cases is important when discussing potential outcomes with patients and weighing the risks and benefits of surgical intervention in this challenging and heterogeneous population. Further case reports, both positive and negative, ideally incorporating neurophysiological outcomes and VTA modeling, may help bridge this knowledge gap.

## Additional File

The additional file for this article can be found as follows:

10.5334/tohm.1171.s1 Supplementary File. DBS programming parameters and Patient Videos.
