# Supplementary material for: Post-Hypoxic Myoclonus and Deep Brain Stimulation. Experience from a Small Patient Cohort and Literature Review Highlighting Variable Outcomes
Source: Tremor Other Hyperkinet Mov (N Y). 2026 Jul 7;16:43. doi: 10.5334/tohm.1171 (PMC13353099; doi:10.5334/tohm.1171)
Supplement: Supplementary File. — DBS programming parameters and Patient Videos. [file tohm-16-1-1171-s1.zip › tohm-1171_pourfar-s1/tohm-1171_pourfar-Supplementary+file+2.27.2026.docx]

Search strategy

A Medline search was conducted using the following search strategy: (hypox*[TiAb]OR anox*[TiAb]) AND (myoclon*[TiAb] OR Lance Adams[TiAb] ) AND (DBS[TiAb]OR Deep brain Stimulation[TiAb] )

There were no date limits, both searches were updated to October 2025 and restricted to English. Initial search results were screened by checking the title and abstract; Full-text articles from the resultant list were evaluated for inclusion. Supplementary Figure 1 reports the selection flow chart. We subsequently reviewed the articles’ references and reviewed papers to identify suitable reports not populated by the keyword-based search strings.


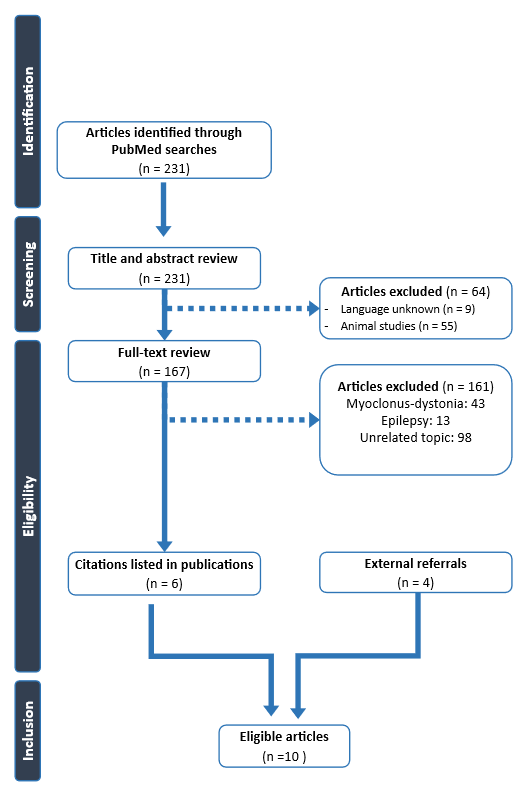


**Supplementary figure 1: PRISMA flow diagram of the different phases of the search strategy. It maps out the number of identified records, included and excluded, and the reasons for exclusion**

Supplementary tables

| **DOS** | **Initial programming** | **Visit1** | **Visit 2** | **Visit 3** | **Visit 4** | **Visit 5** | **Visit 6** | **Visit 7** | **Visit 8** |
| --- | --- | --- | --- | --- | --- | --- | --- | --- | --- |
| 6/29/2020 | 6/30/2020 | 8/21/2020 | 11/20/2020 | 3/5/2021 | 6/21/2020 | 8/3/2021 | 9/10/2022 | 6/12/2023 | 10/03/2023 |
| Stimulation  parameters | **L: C+2-: 1.0/90/130**  **R: 10+11- 1.0/60/90** | L C+2-: 4.0/90/130, R: 10+11-: 3.0/90/130 | L: 3+2-1-: 2.0/130/30  R: 10+11-: 2.0/130/30 | L: 3+2-1-: 2.0/130/30  R: 10+11-: 2.5/130/30 | L: 3+2-1-: 2.0/300/32  R: 10+11-:2.0/300/32 | L: 3+2-1-: 2.0/300/32  R: 10+11-: 2.0/300/32 | L: 3+2-1-: 3.5/230/32  R: 10+11-: 10+11-: 2.8/300/32 | L: 3+2-1-: 3.7/230/32  R: 10+11-: 3.7/300/32 | L: 3+1-2- 3.7/230/32 R: 10+11- 3.70/300/32 |
| Medications |  | Clonazepam 0.5 mg BID  Valproate  1,000+1000  Levetiracetam 1500 BID  Zonisamide 200 mg BID | Levetiracetam 1000 mg BID  Clonazepam 2 mg QID  Valproate 1000 mg BID | Levetiracetam 1,000 mg BID  Clonazepam 2 mg QID  Valproate 1000 mg  BID  Zonisamide 200 mg BID | Levetiracetam 1000mg BID  Clonazepam 0.5 mg 1.5mg QID  Valproate 750mg BID  Baclofen pump | Levetiracetam 1000mg BID  Clonazepam 2 mg BID  Valproate 1000 +750mg | Clonazepam 1.5mg 3x+2mg  Valproate 1000+750  Levetiracetam1250+1250 | Valproate ER 250 mg BID  Clonazepam 1.5mg 3x+2mg  Levetiracetam, 1250 BID  Primidone  50 mg | Valproate ER 250 mg BID  Clonazepam  1.5mg 3x+2mg  Levetiracetam 1250 BID |

**Supplementary Table 1: DBS parameters of patient 1 at each follow-up visit Abbreviations:** BID: two times daily, L: left; QID: four times daily, R: right; TID: three times daily

| **DOS** | **Initial programming** | **Visit 2** | **Visit 3** | **Visit 4** |
| --- | --- | --- | --- | --- |
| 2/10/21 | 2/23/2021 | 7/14/2021 | 10/31/2023 | 4/2/2025 |
| Stimulation parameters | L: C+2- 1.5/60/130  R: C+11- 1.5/60/130 | L: C+2-  3.0/90/130  R: C+11- 3.0/90/130 | L: C+2- 2.7/100/130  R:C+11- 2.7/100/130 | R: 12+11- 2.75/90/130  L: 4+3- 2.75/90/130 |
| Medications | Sodium Oxybate 2g TID  Levetiracetam 1500mg BID-Zonisamide 100mg BID  Clonazepam 0.5mg BID | Sodium Oxybate 2 g  TID  Valproate ER 750mg BID  Levetiracetam 750mg 1 BID  Zonisamide 100mg BID  Clonazepam 0.5mg QID | Sodium Oxybate 2g TID  Valproate ER 750mg BID  Levetiracetam 750mg 1 BID  Zonisamide 100mg BID  Clonazepam 0.5mg QID | Perampanel 2 mg QID  Valproate ER 250mg TID  Keppra 750 mg BID |

**Supplementary Table 2: DBS parameters of patient 2 at each follow-up visit**

**Abbreviations:** BID: two times daily, L: left; QID: four times daily, R: right; TID: three times daily

| **DOS** | **Initial programming** | **Visit 2** | **Visit 3** | **Visit 4** |
| --- | --- | --- | --- | --- |
| 8/27/2018 | 9/21/2018 | 11/7/2018 | 2/11/2019 | 9/10/2021 |
| Stimulation parameters | L: 0-1+: 2.0V/ 90/130  R: 8-9+: 2.0V/90/130 Hz  Program B  L: 1-2-3+:  2.0V/ 90/130  R: 9-10-11+: 2.0V/ 90/130 | L: 1+0- 3.0/90/130  R: 9+8- 3.0/90/130 | L: C+1- 2.5/60/130  R: C+9- 2.5/60/130 | L: C+1- 1.0/130/30  R: 8+9- 1.0/130/30 |
| Medications | Perampanel 4 mg  Sodium Oxybate 4ml QID,  Clonazepam 2.5 mg  Valproate 1000 mg BID  Levetiracetam 1000 mg BID | Perampanel 2 mg  Sodium Oxybate  4ml QID,  Clonazepam 2.5 mg  Valproate 1000 mg BID  Levetiracetam 1000 mg BID | Ativan 1mg  Perampanel 2 mg,  Xyrem 4ml QID,  Clonazepam 2.5 mg  Valproate 1000 mg BID  Levetiracetam 1000 mgBID | Briviracetam 100 mg BID  Lacosamide 1000 TID Perampanel 2 mg  Clonazepam 2mg TID  Valproate 500 mg TID |

**Supplementary Table 3: DBS parameters of patient 3 at each follow-up visit**

**Abbreviations:** BID: two times daily, L: left; QID: four times daily, R: right; TID: three times daily

| **Study** | **Neurophysiology** |
| --- | --- |
|  |  |
| **Kobayashi et al.^1^** | Electromyography using surface  electrodes revealed irregular and repetitive burst discharges when the patient performed any action, particularly elevation of the arm or holding a cup |
| **Yamada et al. ^2^** | EEG showed no electrographic correlate |
| **Asahi et al. ^3^** | Surface electromyograms revealed a cortical myoclonus pattern. Giant somatosensory-evoked potentials were not observed. |
| **Ramdhani et. al ^4^** | EEG monitoring did not reveal seizure activity |
| **Gao et al. ^5^** | EEG showed no electrographic correlates |
| **Mure et al.^6^** | A resting EEG demonstrated a generalized, slow activity in her reference electrodes without any epileptic discharges.  Giant somatosensory evoked potentials not present. |
| **Kim et al.^7^** | EEG findings indicated generalized cortical myoclonus. |
| **Ozturk et al.^8^** | EEG did not reveal any seizure activity |
| **Tharp et al.2024^9^** | The stereotactic EEG showed that his bilateral tonic-clonic seizures originated from the mid primary [sensory cortex](https://www.sciencedirect.com/topics/medicine-and-dentistry/sensory-cortex) with secondary generalization and that his myoclonus was cortical in nature, with the left arm myoclonic jerks associated with [ictal](https://www.sciencedirect.com/topics/medicine-and-dentistry/ictal) spikes in the right [primary motor cortex](https://www.sciencedirect.com/topics/medicine-and-dentistry/primary-motor-cortex) and the right arm myoclonic jerks associated with the left primary [motor cortex](https://www.sciencedirect.com/topics/agricultural-and-biological-sciences/motor-cortex). |
| **Kaur^10^** | EEG showed frequent myoclonic jerks without ictal correlate and generalized epileptiform discharges that are occasionally associated with myoclonic jerks. |

**Supplementary table 4: Neurophysiological findings from previous studies**

Supplementary video

**Segment 1 (0:00–0:21):** Pre-operative condition of Patient 1. The video shows action-induced myoclonus and resting myoclonus in all the four limbs, more prominent in the lower limbs, and negative myoclonus interfering with the ability to stand unassisted and to walk.

**Segment 2 (0:22–0:45):** Post-operative condition of Patient 1. The patient could stand more easily for a period of time despite persistent negative myoclonus and was unable to walk.

**Segment 3 (0:46–1:12):** Pre-operative condition of Patient 2. The video shows axial myoclonus at rest and intermittent negative myoclonus. The patient was able to stand and walk with assistance from one person.

**Segment 4 (1:13–1:30):** Post-operative condition of Patient 2. The video shows persistent intermittent action myoclonus of the upper limbs in the outstretched position. The patient was able to stand unassisted but refused to walk without a walker due to gait instability.


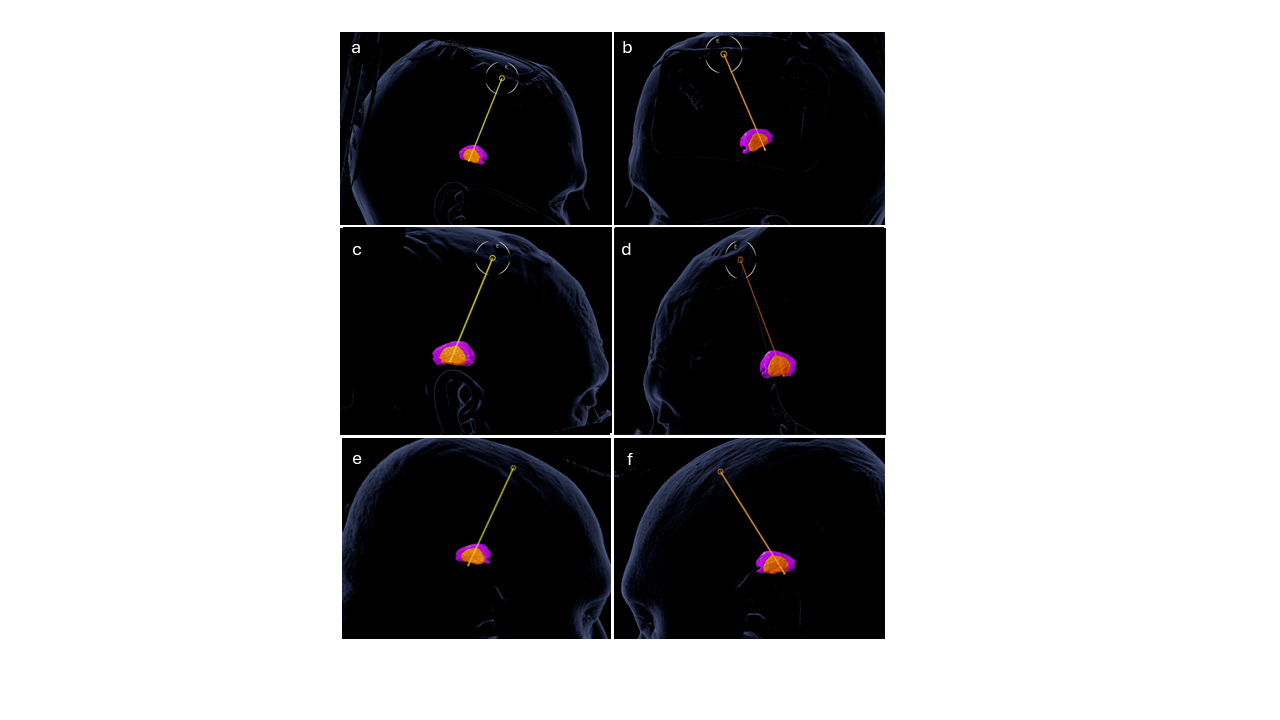


**Supplementary figure 2**: neuroanatomical images for each patient’s leads, showing the lead, GPi (in orange), and GPe (in purple). **Patient 1:** a, left; b, right. **Patient 2:** c, left; d, right. **Patient 3:** e, left; f, right.

References

1. Kobayashi K, Katayama Y, Otaka T, et al. Thalamic deep brain stimulation for the treatment of action myoclonus caused by perinatal anoxia. *Stereotact Funct Neurosurg* 2010; **88**(4): 259-63.

2. Yamada K, Sakurama T, Soyama N, Kuratsu J. Gpi Pallidal Stimulation for Lance-Adams Syndrome. *Neurology* 2011; **76**(14): 1270-+.

3. Asahi T, Kashiwazaki D, Dougu N, et al. Alleviation of myoclonus after bilateral pallidal deep brain stimulation for Lance-Adams syndrome. *J Neurol* 2015; **262**(6): 1581-3.

4. Ramdhani RA, Frucht SJ, Kopell BH. Improvement of Post-hypoxic Myoclonus with Bilateral Pallidal Deep Brain Stimulation: A Case Report and Review of the Literature. *Tremor Other Hyperk* 2017; **7**.

5. Gao F, Ostrem JL, Wang DD. Treatment of Post-Hypoxic Myoclonus using Pallidal Deep Brain Stimulation Placed Using Interventional MRI Methods. *Tremor Other Hyperkinet Mov (N Y)* 2020; **10**: 42.

6. Mure H, Toyoda N, Morigaki R, Fujita K, Takagi Y. Clinical Outcome and Intraoperative Neurophysiology of the Lance-Adams Syndrome Treated with Bilateral Deep Brain Stimulation of the Globus Pallidus Internus: A Case Report and Review of the Literature. *Stereotact Funct Neurosurg* 2020; **98**(6): 399-403.

7. Kim MJ, Park SH, Heo K, Chang JW, Kim JI, Chang WS. Functional Neural Changes after Low-Frequency Bilateral Globus Pallidus Internus Deep Brain Stimulation for Post-Hypoxic Cortical Myoclonus: Voxel-Based Subtraction Analysis of Serial Positron Emission. *Brain Sci* 2020; **10**(10).

8. Öztürk. Is deep brain stimulation useful in Lance–Adams

syndrome? *Neurology Asia* 2021; **26**: 617 – 20.

9. Tharp E, Hafeez MU, Gavvala J, et al. Treatment of refractory post-hypoxic myoclonus and focal epilepsy with subthalamic nuclei deep brain stimulation. *Parkinsonism Relat Disord* 2024; **127**: 107056.

10. Kaur H, Goble TJ, Fenoy A, Ramdhani RA. Deep Brain Stimulation for Post-Hypoxic Myoclonus: A Case Correlating Local Field Potentials to Clinical Outcome. *Tremor Other Hyperkinet Mov (N Y)* 2025; **15**: 16.
